# Inhibition of EGFR/MAPK signaling reduces microglial inflammatory response and the associated secondary damage in rats after spinal cord injury

**DOI:** 10.1186/1742-2094-9-178

**Published:** 2012-07-23

**Authors:** Wen-sheng Qu, Dai-shi Tian, Zhi-bao Guo, Jun Fang, Qiang Zhang, Zhi-yuan Yu, Min-jie Xie, Hua-qiu Zhang, Jia-gao Lü, Wei Wang

**Affiliations:** 1 Department of Neurology, Tongji Hospital, Tongji Medical College, Huazhong University of Science and Technology, 1095 Jiefang Avenue, Wuhan, 430030, China; 2 Department of Neurosurgery, Tongji Hospital, Tongji Medical College, Huazhong University of Science and Technology, 1095 Jiefang Avenue, Wuhan, 430030, China; 3 Department of Cardiovascular Medicine, Tongji Hospital, Tongji Medical College, Huazhong University of Science and Technology, 1095 Jiefang Avenue, Wuhan, 430030, China

**Keywords:** Epidermal growth factor receptor, Microglia, Spinal cord injury, Neuroinflammation

## Abstract

**Background:**

Emerging evidence indicates that reactive microglia-initiated inflammatory responses are responsible for secondary damage after primary traumatic spinal cord injury (SCI); epidermal growth factor receptor (EGFR) signaling may be involved in cell activation. In this report, we investigate the influence of EGFR signaling inhibition on microglia activation, proinflammatory cytokine production, and the neuronal microenvironment after SCI.

**Methods:**

Lipopolysaccharide-treated primary microglia/BV2 line cells and SCI rats were used as model systems. Both C225 and AG1478 were used to inhibit EGFR signaling activation. Cell activation and EGFR phosphorylation were observed after fluorescent staining and western blot. Production of interleukin-1beta (IL-1β) and tumor necrosis factor alpha (TNFα) was tested by reverse transcription PCR and ELISA. Western blot was performed to semi-quantify the expression of EGFR/phospho-EGFR, and phosphorylation of Erk, JNK and p38 mitogen-activated protein kinases (MAPK). Wet-dry weight was compared to show tissue edema. Finally, axonal tracing and functional scoring were performed to show recovery of rats.

**Results:**

EGFR phosphorylation was found to parallel microglia activation, while EGFR blockade inhibited activation-associated cell morphological changes and production of IL-1β and TNFα. EGFR blockade significantly downregulated the elevated MAPK activation after cell activation; selective MAPK inhibitors depressed production of cytokines to a certain degree, suggesting that MAPK mediates the depression of microglia activation brought about by EGFR inhibitors. Subsequently, seven-day continual infusion of C225 or AG1478 in rats: reduced the expression of phospho-EGFR, phosphorylation of Erk and p38 MAPK, and production of IL-1β and TNFα; lessened neuroinflammation-associated secondary damage, like microglia/astrocyte activation, tissue edema and glial scar/cavity formation; and enhanced axonal outgrowth and functional recovery.

**Conclusions:**

These findings indicate that inhibition of EGFR/MAPK suppresses microglia activation and associated cytokine production; reduces neuroinflammation-associated secondary damage, thus provides neuroprotection to SCI rats, suggesting that EGFR may be a therapeutic target, and C225 and AG1478 have potential for use in SCI treatment.

## Backgrounds

The pathophysiology of traumatic spinal cord injury (SCI) is thought to include two stages [1]. As primary insult, the direct mechanical damage cannot be therapeutically influenced. However, secondary damage, including electrolyte abnormalities, free radical formation, vascular ischemia, edema, posttraumatic inflammatory reaction, apoptosis and other processes, are amenable to various therapeutic interventions. In particular, reports emphasizing the importance of the inflammatory process in mediating tissue damage after SCI have been accumulating [2-4]. Although inflammation is a physiological response to injury, evidence suggests that early inflammatory changes after SCI are detrimental, as reviewed by [5], leading to glial scar formation, neuronal loss and demyelination, ultimately worsening outcomes.

Microglia are a prominent source of inflammatory mediators; these cells undergo profound activation in response to injury (reviewed by [6]). They constantly survey the microenvironment for noxious agents and injurious processes, respond to extracellular signals, clean cellular debris and toxic substances, and secret trophic factors, thereby providing neuroprotection after central nervous system (CNS) injury. On the other hand, activation of microglia, with resultant production of proinflammatory mediators and neurotoxic molecules, is involved in the spread of secondary injury. There is mounting evidence that microglia activation is one of the major causes of secondary damage after SCI, and that suppressing it can reduce tissue damage and improve morphological/functional recovery [7-9].

Modulating the microglial inflammatory process might create a niche environment for tissue repair. Recently, a well-documented receptor, epidermal growth factor receptor (EGFR), attracted much attention for its potency in regulating cell activation. Binding of ligands like EGF and tumor necrosis factor α (TNFα), the tyrosine-specific protein kinase intrinsic to EGFR, results in activation, and is followed by transactivation of mitogen-activated protein kinase (MAPK) and other downstream signal pathways [10]. The activation of MAPK has been reported to be essential for production of several inflammatory cytokines, such as interleukin (IL)-1β, TNFα and IL-6 [11,12]. In the CNS, EGFR localizes in neurons, astrocytes, and oligodendrocytes, as well as in microglia [13,14]. Activation of EGFR was reported to cause formation of cribriform structures in astrocytes, related to guided migration [15]. EGFR mediates the EGF-induced chemotactic and chemokinetic migration of microglia [16], and EGFR signaling functions in several CNS disorders, such as ischemia [17], tuberous sclerosis [18], and Alzheimer’s disease [19], as well as after SCI [14].

Therefore, we hypothesized that regulation of EGFR signaling may influence activation of microglia and associated neuroinflammation, thus attenuating secondary damage after SCI. In the present study, lipopolysaccharide (LPS)-activated microglia cultures and traumatic SCI rats were used as model systems to observe phosphorylated EGFR (pEGFR) expression, microglia activation, cytokine production, morphological and functional outcomes, as well as the underlying mechanisms resulting after EGFR blockade by C225 and AG1478, a blocking antibody and a specific tyrosine kinase inhibitor, respectively [20,21].

## Methods

Detailed information of reagents has been provided in Additional file 1.

### Surgical procedures and reagent delivery

All experimental procedures were performed in accordance with protocols approved by the Governmental Animal Care Committee of Tongji Medical College. During surgery, rats were placed on a warming pad to maintain body temperature of 37.0 ± 0.5°C. After injury, animals were returned to individual cages with sufficient water and food; each received a daily penicillin injection (200,000 U per rat, intramuscular) for three days.

Adult Wistar male rats (240 to 260 g weight) were randomly assigned into four experimental groups: sham-operated, SCI-induced, C225-treated, and AG1478-treated. Traumatic SCI was induced by the weight-drop technique, as described previously [14]. Briefly, rats were anesthetized with intraperitoneal ketamine (75 mg/kg) and xylazine (20 mg/kg) injections. A T11 spinal laminectomy was made to expose spinal cord, and a moderate intensity weight drop (10 g × 12.5 cm) was performed by MASCIS Impactor II (New York University, USA). Rats in the sham-operated group underwent similar procedures as the SCI-induced group, expect for the weight-drop step; rats in both groups were treated with saline through pumps by the following method.

Immediately after SCI induction, a subcutaneous osmotic pump (Model 2002, Alzet, Cupertino, CA, USA) was placed closely to the injury site for intrathecal reagent infusion. Before plantation, the pumps had been filled with 200 μl saline, C225 (2 μM) or AG1478 (1 mM), connected to a 1.5 mm long PE-10 tube, then preincubated overnight at 37°C. On day 7 after infusion, the pump was removed and the wound was closed with sutures.

### Cell culture

Highly purified primary microglia cultures were prepared using modified methods [22]. Briefly, spinal cords of newborn Wistar pups were dissociated, and cells were carried in mixed cultures for two days. Medium was then refreshed with high glucose DMEM containing 20% fetal bovine serum. Ten days later, microglia cells were isolated by an orbital shaker (37°C, 200 rpm, 2 h). Half an hour after cell implantation, the medium was refreshed for further purification. After identification with CD11b antibody, cultures more than 97% pure were used for experiments.

Since a limited number of primary culture cells are available, BV2 cells (from the Chinese Academy of Medical Science) were used as succedaneum for western blot analysis. BV2 is an immortalized microglia cell line that is reported to share many characteristics with primary microglia [23,24]. The cells were cultured in fresh high glucose DMEM supplemented with 10% fetal bovine serum at a density not exceeding 5 × 10^5^ cells/cm^2^.

### Western blot analysis

After sacrifice under deep anesthesia by transcardial saline infusion, experimental rat spinal cord tissue (1.5 mm long, centered at the injury site) was quickly removed and homogenized by sonication in RIPA lysis buffer. Similarly, cultured cells were lysed by RIPA, scraped off and collected for protein extraction. Lysates were centrifuged at 12,000 x g at 4°C for 10 min and supernatants were collected for the protein concentration assay, performed using a BCA kit.

Samples containing 60 μg total protein were loaded on SDS-PAGE (10% for EGFR/pEGFR; 12% for other components), and then transferred to nitrocellulose membranes (300 mA, 4 h for EGFR/pEGFR; 250 mA, 2 h for others). After blocking nonspecific binding, blots were incubated with primary antibody overnight at 4°C, followed by conjugation with horseradish peroxidase (HRP)-conjugated immunoglobulin G (IgG). Finally, blots were visualized with enhanced chemiluminescence (ECL) kits, and resulting digital images were analyzed by Image J (National Institute of Health, Bethesda, MD, USA) to obtain the optical densities (OD) of signals. OD of tested proteins was normalized to OD of β-actin; the gained ratio was normalized with its corresponding control (taken as 100%); finally, statistical comparison was performed and results were expressed as diagrams (most were shown as Additional file 1).

### Cell treatment and experimental tests

Cells were seeded at 1 × 10^5^ cells/cm^2^ onto glass coverslips in 24-well culture plates. Inhibitors were given 30 min before LPS (1 μg/ml) stimulation, with final concentrations at 20nM (C225) or 10 μM (AG1478/U0126/SB203580/SP600125). The solvent served as the control treatment. Supernatants were collected for ELISA, while cells were fixed by methanol (−20°C, 15 min) for staining at various harvesting time points.

Concentrations of IL-1β and TNFα were measured by ELISA according to the manufacturer’s protocol. For double-staining, fixed cells were blocked with 5% BSA/PBS at 20 ± 2°C for 1 h, incubated simultaneously with CD11b and pEGFR antibody at 4°C for 16 h, incubated with corresponding fluorescent conjugated anti-IgG at 20 ± 2°C for 1 h, then labeled with DAPI. Finally, the coverslips were examined at multiple sites under a laser scanning confocal microscope (LSM710, Carl Zeiss, Germany). To evaluate cell hypertrophy, somata size of microglias was semi-quantified according to reported method [25]. Briefly, Image J software was used to calculate surface areas of CD11b^+^ cells. At least 20 cells were randomly collected in each sample, and the averaged area was taken for statistical analysis.

For reverse transcriptase-PCR, cells were cultured in 12-well plates and the total mRNA was extracted using MagExtractor (Toyobo, Osaka, Japan). One ug mRNA was reverse transcribed with ReverTra Ace (Toyobo, Osaka, Japan). Subsequent PCR reactions were performed with the hot-start PCR mix with a 25 μl reaction volume, taking 1 μl cDNA as a template. Detailed PCR procedure has been provided in Additional file 1. After electrophoresis, images were processed using a Gene Genius Bio-Imaging system (Syngene, Frederick, MD, USA). Target gene expression was normalized versus the housekeeping gene glyceraldehyde 3-phosphate dehydrogenase (GAPDH) using OD ratios; then, normalized with its corresponding control (taken as 100%); finally, statistical comparison was performed and results were expressed as Additional file 1.

### Tissue processing, staining and edema analysis

Anesthetized rats were transcardially infused with saline, followed by ice-cold Zamboni’s fixative. Spinal cord tissues containing the injury site were extracted, fixed for 24 h in Zamboni’s fixative, cryoprotected in 30% sucrose/0.1 M PBS for three days at 4°C, and finally cut longitudinally into 30 μm sections for fluorescent staining.

Briefly, sections were incubated with primary antibody for 16 h at 4°C, conjugated with corresponding secondary antibody for 1 h at 20 ± 2°C, then observed under a microscope (Carl Zeiss). Four sections taken at 0.5 mm intervals in the spinal cord were stained, four fields at pertinent sites were captured.

Spinal cord edema was evaluated by determining the water content [26]. After sacrifice, spinal cord tissue (1.5 mm long, centered at the injury site) was quickly removed and weighed precisely (wet weight). Then the tissue was dried for 48 h at 80°C to determinate the dry weight. Water content = (wet weight − dry weight)/wet weight × 100%.

### Anterograde tracing and behavioral measurement

According to a method described previously [27], 10% biotinylated dextran amine (BDA) was injected into the right side of the spinal cord through the T7-T8 intervertebral space 28 d post-SCI. A total of 1 μl BDA was injected by micropipette with a 0.2 mm diameter tip, at depths of 0.5 and 1.0 mm, 1.0 mm lateral to the dorsal median sulcus; four days later, rats were anesthetized and fixed with Zamboni’s fixative, as described above. Horizontal 30 μm sections were incubated with fluorescein isothiocyanate (FITC)-avidin, followed by glial fibrillary acidic protein (GFAP) visualization with primary antibody and cyanine 3 (Cy3)-conjugated IgG. Labeled tissues were captured under a 10× objective, and photos were reorganized using Photoshop CS 8.0 (Adobe System Incorporated, San Jose, CA, USA).

Behavioral outcome was evaluated strictly according to two scales, Basso, Beattie and Bresnahan (BBB) [28] and combined behavior score (CBS) [29], at days 1, 7, 14 and 28 after SCI, by two trained investigators blinded to the experimental groups.

### Statistical analysis

After a simple randomization, seven rats in each group were used for behavioral observation, while for other protocols five samples were used. All the descriptions about significant difference are based on statistic analysis, while figures for statistic comparison are added as supplementary materials. Statistical difference between groups (defined as *P* < 0.05) was evaluated with one-way ANOVA followed by Tukey’s post hoc test. Data are presented as mean ± SE.

## Results

### EGFR blockade inhibits LPS-induced microglia activation and EGFR phosphorylation

**Figure 1:**
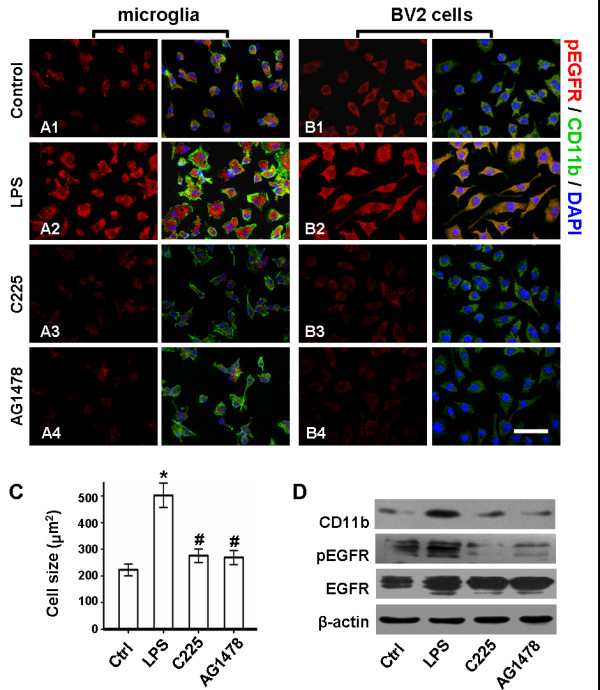
**EGFR blockade inhibits LPS-induced microglia activation and EGFR phosphorylation.** Plated cells were treated with 20nM C225 or 10 μM AG1478 30 min before 1 μg/ml LPS stimulation. After 3 h LPS stimulation, co-staining of CD11b (Green), pEGFR (Red) and nucleus (Blue) was performed. pEGFR expressed in microglias (**A1**) and BV2 cells (**B1**) is elevated after LPS stimulation (**A2/B2**); C225 (**A3/B3**) or AG1478 (**A4/B4**) inhibits LPS-induced overexpression of pEGFR. Scale bar = 50 μm. (**C**) Comparison of cell size suggests that the microglial hypertrophy led by LPS is reduced by C225 or AG1478. *P < 0.01 versus sham; ^#^*P* < 0.01 versus LPS-treated. (**D**) Western blot analysis of BV2 cells reveals that the LPS-induced upregulation of CD11b and pEGFR is effectively reduced by C225 or AG1478. n = 5. LPS, lipopolysaccharide; pEGFR, phosphorylated epidermal growth factor receptor.

It was reported that activated primary microglia are enlarged and have many microspikes covering cell body surfaces, and display intense immunoreactivity due to CD11b antigen, as compared to resting microglia with small, amoeboid shapes [30]. In the present study, such typical changes have been observed after 3 h LPS stimulation to primary microglia (Figure 1A2). Compared with control (223.00 cm^2^ ± 21.95 cm^2^), a 2.25-fold increase of cell size in LPS-treated group (502.80 cm^2^ ± 45.64 cm^2^) suggested the hypertrophy of reactive microglias (Figure 1C). In parallel with morphological activation, immunoreactivity of pEGFR increased (Figure 1A2), whereas this was weak in both membrane and cytoplasm of control cells (Figure 1A1). Similarly, in BV2 cells, reactive changes and elevated pEGFR expression were reflected by fluorescent staining (Figure 1B2), while enhanced expression of CD11b and pEGFR was detected by western blot analysis (Figure 1D).

However, after either C225 or AG1478 pretreatment, the following were observed: prevention of the LPS-induced hypertrophy of primary microglia, resulting in an average cell size of 276.09 ± 25.53 cm^2^ and 269.25 ± 26.24 cm^2^, respectively (Figure 1C); inhibition of EGFR phosphorylation in primary microglias (Figure 1A3 and A4) and BV2 cells (Figure 1B3 and 1B4), confirmed by fluorescent staining; and reduced expression of CD11b and pEGFR in BV2 cells, demonstrated by western blot analysis (Figure 1D).

### EGFR blockade reduces LPS-induced cytokine production in microglia

**Figure 2:**
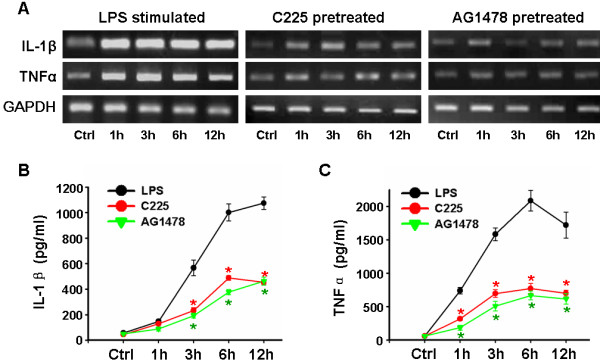
**EGFR blockade depresses LPS-induced cytokine production in microglia.** Purified microglia was treated with 20nM C225 or 10 μM AG1478 30 min before 1 μg/ml LPS stimulation. (**A**) Reverse transcriptase-PCR (cells) and (**B**) ELISA (supernatants) at various time-points. It demonstrates that C225 and AG1478 depress LPS-induced synthesis/secretion of IL-1β and TNFα. n = 5. *P < 0.01 versus LPS-treated. EGFR, epidermal growth factor receptor; LPS, lipopolysaccharide.

Activated microglia produce an array of proinflammatory factors that are key mediators of neuronal inflammation, including IL-1β and TNFα [6]. In the present study, reverse transcriptase-PCR revealed that microglial mRNA expression was markedly upregulated after 1 h LPS stimulation (Figure 2A). Although low in control, secretion of IL-1β and TNFα was dramatically enhanced by LPS, to dozens of times the level found in vehicle-treated groups (57.00 ± 9.18 pg/ml for IL-1β and 59.6 0 ± 11.59 pg/ml for TNFα); a typical result included peaks of (1074.40 ± 48.76) pg/ml IL-1β 12 h, and (2086.00 ± 155.52) pg/ml TNFα 6 h, after LPS stimulation (Figure 2 B and C).

Pretreatment with either C225 or AG1478 depressed this elevation of both mRNA level (Figure 2A) and protein secretion (Figure 2 B and C). For example, in C225-treated groups, IL-1β in the supernatant collected after 12 h of LPS stimulation was (454.40 ± 44.02) pg/ml, while TNFα was (771.60 ± 74.04) pg/ml after 6 h. No statistically significant difference was found between the changes induced by C225 and AG1478, suggesting that equivalence existed.

### MAPK mediates the depression of cytokine production after EGFR blockade

**Figure 3:**
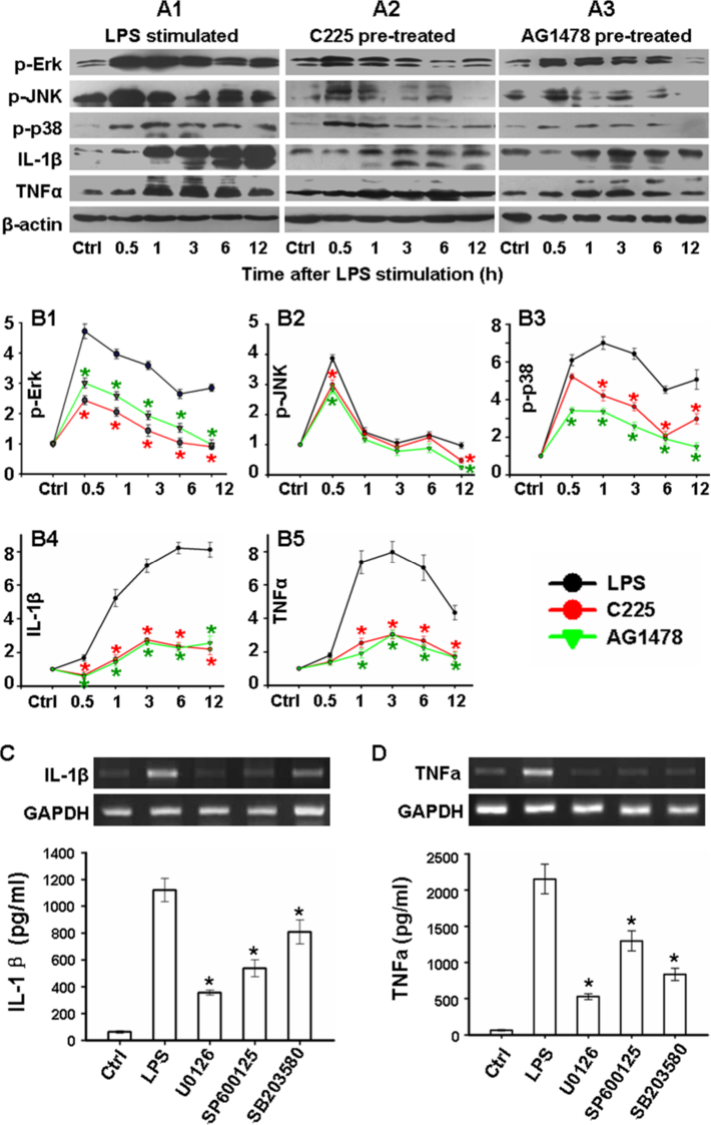
**MAPK signaling mediates the depression of cytokine production by EGFR blockade.** After treated with 20nM C225 or 10 μM AG1478 30 min before 1 μg/ml LPS stimulation, western blot analysis of BV2 cells was performed to show phosphorylation of the MAPKs (Erk, JNK and p38) and cytokine production (IL-1β and TNFα). (**A**) representative photos. (**B**) Statistical comparison after normalization to β-actin and its corresponding control. Primary microglia was pretreated with 10 μM selective MAPK inhibitors (SB203580 for p38, U0126 for Erk and SP600125 for JNK) 30 min before LPS stimulation. Synthesis and secretion of cytokines were tested at 3 h and 6 h after LPS stimulation, respectively. (**C**) and (**D**) show the representative mRNA expression and analyzed supernatant protein concentrations of IL-1β and TNFα, respectively. n = 5. *P < 0.01 versus LPS-treated. EGFR, epidermal growth factor receptor; Erk, extracellular signal-regulated kinases; JNK, c-jun terminal kinase; LPS, lipopolysaccharide; MAPK, mitogen-activated protein kinases.

All three major members of the MAPK family - extracellular signal-regulated kinases (Erk), c-jun terminal kinase (JNK) and p38 - have been reported to be responsible for cytokine production [12,31,32]. In this study, after treatment with LPS, temporal activation in BV2 cells of each MAPK type was detected by western blot. It was demonstrated that LPS stimulation resulted in a rapid phosphorylation, within 0.5 h, of Erk, JNK and p38, and prolonged phosphorylation of Erk and p38, up to 12 h after stimulation (Figure 3A1, B1-3).

Expression of IL-1β and TNFα was also determined in BV2 cells. IL-1β was progressively upregulated during 12 h observation after LPS stimulation (Figure 3A1 and B4). Accompanying the elevation of pro-IL-1β (31 kDa), the mature secretory form (17.5 kDa) was synchronously increased. Similarly, both membrane and soluble forms of TNFα (26 kDa and 17 kDa respectively) were elevated at 1 h, peaking 3 h after LPS stimulation (Figure 3A1 and B5). The chronological order of these changes suggested that MAPK activation and cytokine production may be correlated.

In order to confirm this hypothesis, primary microglias were pretreated with selective inhibitors of the MAPK pathways (SB203580 for p38, U0126 for Erk and SP600125 for JNK) 30 min before LPS treatment separately; all of which resulted in depressed mRNA expression and secretion of IL-1β/TNFα, to different degrees (Figure 3C and D). U0126 was most effective, resulting in 68.7% inhibition of IL-1β, and 75.4% inhibition of TNFα, secretion.

Considered collectively, these results support the hypothesis that MAPK signaling mediates LPS-induced production of both IL-1β and TNFα. MAPK is also known as a major downstream pathway for EGFR [33,34], thus was also tested it in BV2 cells after C225 and AG1478 treatment here. Both them depressed the phosphorylation of MAPK, especially activation of Erk and p38 (Figure 3A2-3 and B1-3). Consistently, production of IL-1β and TNFα was significantly reduced after C225 and AG1478 treatment of BV2 cells (Figures 3).

### EGFR activation appears in reactive microglia in the early phase following SCI

**Figure 4:**
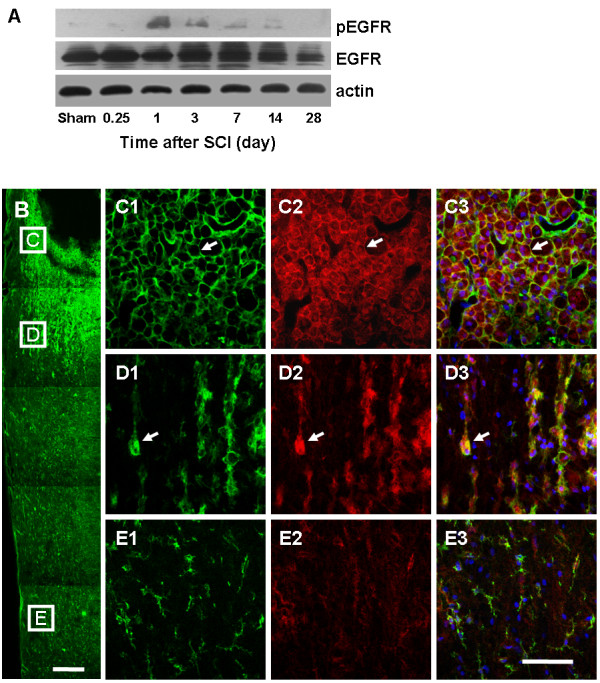
**EGFR phosphorylation is elevated in parallel with microglia activation in early-phase SCI.** (**A**) Representative western blots reveal the elevated expression of pEGFR on days 1 to 14, peaking on day 1. (**B**) Fluorescent staining of CD11b (Green) demonstrates the gradient activation of microglias on day 3 after SCI. Bar = 500 μm. Double staining of pEGFR (Red) and CD11b reveals that pEGFR^+^ reactive microglias surround the cavity (**C**) and appear in the boundary zone (**D**), but not in other areas (**E**). Bar = 100 μm. Arrow, representative pEGFR^+^ reactive cells. pEGFR, phosphorylated epidermal growth factor receptor; SCI, spinal cord injury.

Although limited expression appeared in spinal cords of sham-operated rats, pEGFR was immediately induced and positively expressed on days 1 to 14 after SCI, peaking on day 1, as demonstrated by western blot. Conversely, total EGFR experienced a limited change after the injury (Figure 4A).

EGFR has been reported to be widely expressed in CNS [13,14]. The current study demonstrated that the EGFR phosphorylation is positively related to microglial activation. By double staining, on day 3 after SCI, CD11b^+^ microglias surrounding the cavity or in the boundary zone had reactive morphology and elevated CD11b immunoreactivity, where high expression of membrane pEGFR was located (Figure 4C and D). In contrast, no pEGFR expression was found in resting microglia from remote areas (Figure 4E).

### EGFR blockade reduces EGFR/MAPK activation and cytokine production after SCI

**Figure 5:**
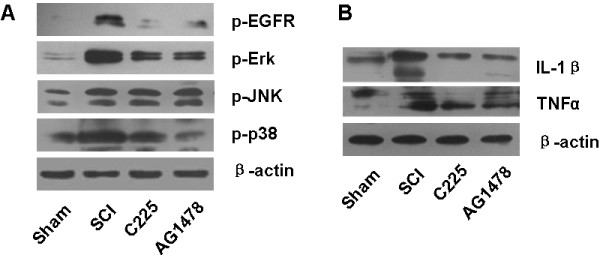
**EGFR blockade reduces EGFR/MAPK activation and cytokine production*in vivo*.** Continual infusion of C225 or AG1478 was performed immediately after SCI (n = 5). Representative photos of western blots reveal (**A**) reduced phosphorylated forms of EGFR, Erk, and p38 on day 1, as well as (**B**) subsequent downregulated expression of IL-1β and TNFα on day 3, after C225 or AG1478 treatment. EGFR, epidermal growth factor receptor; Erk, extracellular signal-regulated kinases; MAPK, mitogen-activated protein kinases.

Continual infusion of either C225 or AG1478 was performed on rats immediately after SCI. To confirm their pharmacological effects *in vivo*, pEGFR expression was examined, and was found to be effectively depressed by the treatments on day 1 after SCI (Figure 5A). In addition, although significantly upregulated by SCI, phosphorylation of Erk and p38 was depressed on day 1 (Figure 5A), while expression of IL-1β and TNFα was reduced on day 3, after SCI (Figure 5B), by either C225 or AG1478 treatment.

### EGFR blockade attenuates secondary damage and contributes to recovery after SCI

**Figure 6:**
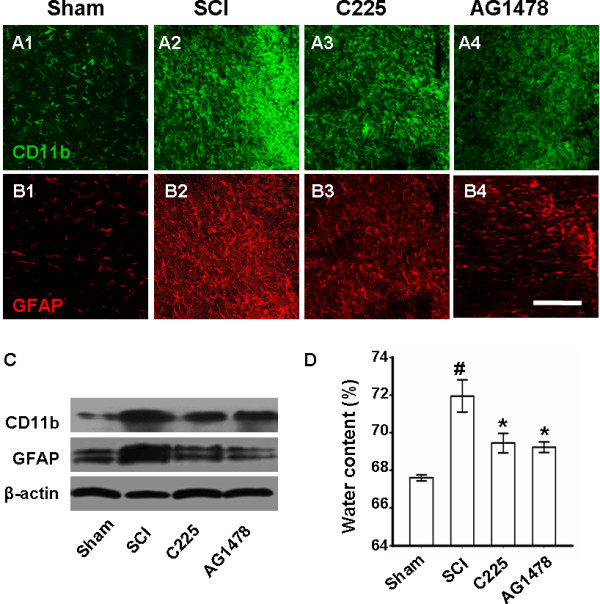
**EGFR blockade attenuates cell activation and tissue edema after SCI.** Fluorescent staining reveals (**A2** and **B2**) the activation of microglia and astrocytes on day 7 after SCI, which was attenuated by (**A3** and **B3**) C225 and (**A4** and **B4**) AG1478 treatments. Bar = 200 μM. (**C**) Similar findings by western blot analysis of CD11b and GFAP 7 day after SCI. (**D**) Water content of spinal cord reveals that SCI-induced edema was reduced by three days C225/AG1478 treatment. n = 5. ^#^*P* < 0.01 versus sham; *P < 0.01 versus SCI. EGFR, epidermal growth factor receptor; GFAP, glial fibrillary acidic protein; SCI, spinal cord injury.

Elevated expression of IL-1β and TNFα was reported to be essential for glial activation and tissue edema [35,36]. In the present study, microglia and astrocyte activation was reflected by elevated expression of CD11b and GFAP on day 7 after SCI (Figure 6A2, B2 and C). Considered together with results of fluorescent staining and western blot analysis, the SCI-induced overexpression of CD11b and GFAP was shown to be attenuated by C225 and AG1478 treatment.

The tissue edema was reflected by water content comparison (Figure 6D). On day 3 after SCI, increased water content was revealed in the SCI group (71.95 ± 0.85%) compared to the sham-operated group (67.60 ± 0.16%); however, this was significantly reduced by either C225 or AG1478 pretreatment (69.45 ± 0.51% and 69.23 ± 0.28%, respectively).

**Figure 7:**
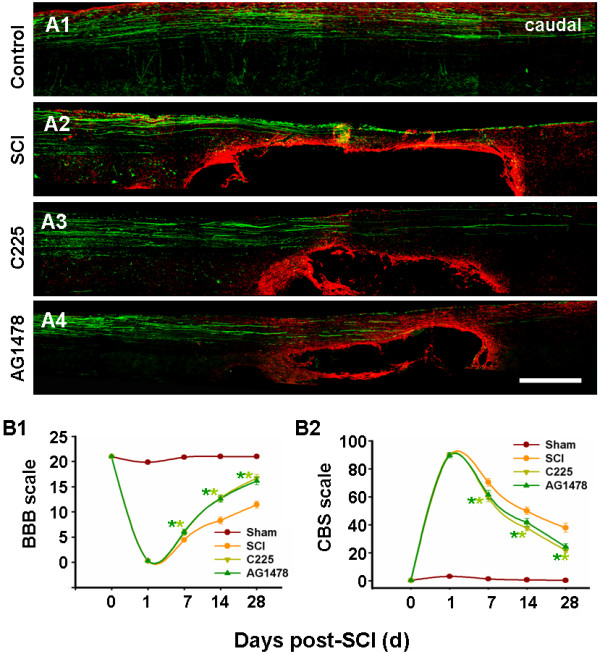
**EGFR blockade promotes morphological and functional recovery.** (**A2**) Combining GFAP staining (red) with BDA tracing (green), fluorescent staining of spinal cord reveals glial scar and cavity formation, as well as limited axonal regeneration after SCI; (**A3** and **A4**) amelioration observed after C225 and AG1478 treatment. Bar = 1 mm. (**B**) Temporal changes in behavioral outcomes, as evaluated by BBB (**B1**) and CBS (**B2**) scoring, indicate that C225 and AG1478 improved functional recovery beginning on day 7 after SCI. n = 7. *P < 0.05 versus SCI. BBB, Basso Beattie and Bresnahan; BDA, biotinylated dextran amine; CBS, combined behavior score; EGFR, epidermal growth factor receptor; GFAP, glial fibrillary acidic protein; SCI, spinal cord injury.

Approximately one month after SCI, anterograde tracing and GFAP staining were applied together to show morphological recovery of damaged rats. As a result, many integrated BDA-labeled fibers and terminals were visualized in sham-operated rats (Figure 7A1); however, few were observed beside or in the caudal side of the injury, and ongoing degeneration was indicated since most axonal end bulbs had formed rostral to the lesion in SCI rats (Figure 7A2). In C225- and AG1478-treated groups, some thin sprouts extended into the nearby gray matter and even appeared caudal to the lesion, although these fibers were shorter in length and branches were fewer in density than those in the sham group (Figure 7A3 and A4).

Reactive astrocytes are the main cell type contributing to the formation of glial scars [37]. In the present study, intense GFAP immunoreactivity was detected around experimental lesions; this was depressed in the C225- and AG1478-treated groups. Cavity formation is considered an important characteristic of SCI damage [1]: in the current study these appeared smaller in the C225- and AG1478-treated groups than in the vehicle-treated group.

Aside from the morphological observations, evaluation of functional recovery was ascertained (Figure 7B), demonstrating the following: that all rats had severe and uniform functional deficits 1 d after SCI; behavioral improvement was observed, but was still incomplete one month after SCI; and, C225 and AG1478 treatment progressively mitigated the functional deficits, with statistical differences seven days after treatment, compared to those in sham-operated rats.

## Discussion

The data presented here provide new insights into the unusual role in microglia activation played by EGFR signaling. This study has demonstrated the following: microglia activation is accompanied by EGFR phosphorylation both *in vitro* and *in vivo*; EGFR blockade by C225 and AG1478 reduces cytokine production in microglia through inhibiting the EGFR/MAPK cascade; and that by modulating the inflammatory response after SCI, inhibition of EGFR signaling reduces activation of microglia and astrocytes, attenuates tissue edema, and finally improves morphological and functional recovery of SCI rats.

As shown in the present study, round or amoeboid-shaped activated microglia form a distinct narrow belt around the lesion by day 7 after SCI. Following SCI, the normally quiescent microglia are inevitably activated, with changes in morphology, motility, proliferation, expression of specific cell surface molecules, and release of cytokines and chemokines, finally becoming so-called ‘reactive microglia’ [38]. Reactive microglia have been considered to be at the center of the injury cascade [4]. Through releasing molecules such as TNFα, IL-1, reactive free radicals and nitric oxide, microglia aggravate early post-injury necrotic cell death, remote cell apoptosis, tissue edema and axonal degeneration [6,39,40].

Therefore, we are persistently trying to modulate microglia activation to improve recovery after SCI. In primary microglia cultures, cell motility, one characteristic of microglia activation, has been reported to be markedly enhanced after EGFR activation [16], which suggests that EGFR is potentially a valuable therapeutic target. *In vitro* and *in vivo*, this study found that activated microglia highly expressed pEGFR, and blocking EGFR activation led to decreased microglia activation and production of IL-1β and TNFα.

Synthesized as a 31 kDa precursor, IL-1β is cleaved to a 17.5 kDa mature form to gain activity; while TNFα is initially expressed as a 26 kDa transmembrane protein, but cleavages to a 17 kDa soluble protein for release. Previous studies have demonstrated the following: IL-1β and TNFα are important proinflammatory factors that mediate changes after SCI [41,42]; infusion of IL-1β into the spinal cord impairs locomotion [43]; and in the acute phase of SCI, TNFα transgenic rats have more spinal cord apoptotic cells than do wild-type rats [41]. What is more, accumulating evidence suggests that moderating production of these factors in early-phase SCI can benefit recovery. For example, blocking IL-1β with receptor antagonists was shown to be useful for counteracting glutamate toxicity and improved morphological and functional recovery [43,44], and inhibition of TNFα either by reagents or antagonist significantly reduced development of inflammation, suppressed neuronal and oligodendroglial apoptosis, facilitated myelin regeneration and improved functional recovery after SCI [45-47].

This study demonstrates that inhibition of EGFR phosphorylation reduces production of IL-1β and TNFα by activated microglia. However, the mechanisms underlying this change remain unclear. Previous reports suggest MAPK signaling pathways might be involved, as follows: 1) the key downstream pathway for LPS-induced signaling events is the MAPK cascade [11]; 2) activation of MAPK was observed to initiate inflammatory responses and aggravated degeneration of neurons in SCI models [48,49]; 3) MAPK is one of the three major downstream pathways for EGFR regulation [33,34]. The present study showed that MAPK was activated by LPS; MAPK inhibitors reduced production of IL-1β and TNFα; in addition, C225 and AG1478 depressed activation of Erk and p38, as well as the expression of IL-1β and TNFα. Considered together, these results suggest that EGFR inhibitors depress inflammation after LPS stimulation and SCI, through regulating the activation of EGFR/MAPK cascade in microglia, which may be a new neuroprotective mechanism after EGFR blockade.

MAPKs are important for intracellular signal transduction and play critical roles in regulating cell proliferation, neural plasticity, inflammatory responses and other biological activities. Previous reports reviewed that p38 and p44/42 MAPKs may play a critical role in harmful microglial activation in acute brain injury [50]; JNK is activated by proinflammatory cytokines and cellular stress, and play essential roles in regulating inflammatory responses [51,52]; activation of MAPK entities, especially Erk and p38, is a determinant of neuronal survival on certain occasions [53-55]; and, selective inhibitors (PD98059 and SB203580) are candidates for treatment [48,49]. We here found that reducing the activation of each MAPK led to the suppression of cytokine production at a different degree, supported by previous reports [32,56]; however, further study is needed to understand the variability between each MAPK signaling.

Secondary damage after SCI is a complicate cascade that involves several immune cell types, including microglia and astrocyte. According to previous reports, activation of microglia is always initialed by proinflammatory factors, and contributes to activation of astrocytes [36,57-59]. We conclude that EGFR blockade may depress cell activation through modulating inflammation, although other mechanisms are possibly operational. For example, astrocytes can be directly activated by EGF through the Rheb-mTOR pathway [60], and the chemotactic migration of microglia was reported to be induced by EGF [16].

Similar to cell activation, the occurrence of tissue edema is a multifactorial process that must include an inflammatory response and disruption of ion regulation and cellular metabolism [35,61]. In the present study, depressed inflammation and cell activation may have ameliorated the altered cellular metabolism and water infiltration after SCI, finally contributing to reduced tissue edema after treatment.

Secondary insults, especially microglia-mediated inflammatory responses and reactive astrogliosis, result in the formation of glial scars and cavities, which have been described as molecular and physical barriers to axonal outgrowth [62]. In contrast to the increased numbers of GFAP-positive astrocytes, large cavity formation and severe axonal damage that appear a month after SCI, in the present study reduced astrogliosis and cavitation, improved axonal growth and functional recovery were observed in the C225- and AG1478-treated groups. It is well known that functional recovery depends on the extent of spared fiber tracts, reorganization of segmental circuitry, and restoration of supraspinal input. Therefore, we presume that through attenuating secondary damage, EGFR blockade provides a beneficial microenvironment for axonal growth, which underlies the subsequent functional improvement. Besides, the wide distribution and multiple functions of EGFR suggest that other mechanisms might underlie the improvement also, for example, regulation of vessel permeability, attenuation of astrogliosis-associated injuries and blockade of the activities of myelin inhibitors [14,63-65].

It is improper to view microglia activation and inflammatory responses as absolutely damaging or beneficial after CNS trauma. Rather the timing for modulation must be considered. Since previous reports suggest that early-phase inflammation is detrimental [5,39,41], we assessed the EGFR regulation in early-phase SCI. Further investigation is needed in order to find the best treatment protocol.

SCI is a catastrophe comprising multiple events. Limitation of methods adopted here results in some imprecise information from animal research, although it can elucidate the observed pathological phenomena more or less. As a newly recognized therapeutic target, regulating EGFR signaling is thought to be neuroprotective. However, negative evidence also exists; for example, EGF was reported to exert a neuroprotective role for the brain after injury [66], and AG1478 promotes CNS axonal growth through certain EGFR-independent processes [67]. Actually, many studies have shown that EGFR can play roles beyond the usual ligand-dependent one, especially after CNS disorders. For example, EGFR can be transactivated after the activation of other membrane receptors, such as angiotensin II receptors [68] and β-2 adrenergic receptors [69]; unpublished results from our group reveal that LPS stimulates phosphorylation of EGFR through enhancing endocelluar calcium activity. Rapid activation of EGFR signaling also occurs after several other CNS disorders, such as electrolytic lesions and entorhinal ablation [70,71], in the damaged brains of patients after stroke, and in those with Alzheimer’s disease [19,72]. Thus, there is a need for further studies into the intricate regulation of EGFR, especially after CNS injury.

## Conclusion

In summary, we report that EGFR signaling is essential for microglia activation and cytokine production, making it a potential therapeutic target for treatment of CNS inflammatory diseases. Rats subjected to spinal cord trauma can be effectively treated with the potent EGFR blockers C225 and AG1478, through modulation of neuroinflammation and associated secondary damage. The fact that EGFR blockers are already used in preclinical research or in clinical settings makes them particularly attractive candidates for clinical trials of SCI treatment modalities.

## Abbreviations

BBB, Basso Beattie and Bresnahan; BCA, bicinchoninic acid; BDA, biotinylated dextran amine; BSA, bovine serum albumin; CBS, combined behavior score; CNS, central nervous system; Cy3, cyanine 3; DAPI, 4,6-diamidino-2-phenylindole; DMEM, Dulbecco’s modified Eagle’s medium; ECL, enhanced chemiluminescence; ELISA, enzyme linked immunosorbent assay; Erk, extracellular signal-regulated kinases; FITC, fluorescein isothiocyanate; GAPDH, glyceraldehyde-3-phosphate dehydrogenase; GFAP, glial fibrillary acidic protein; HRP, horseradish peroxidase; IgG, immunoglobulin; IL, interleukin; JNK, c-jun terminal kinase; LPS, lipopolysaccharide; MAPK, mitogen-activated protein kinases; OD, optical density; PBS, phosphate-buffered saline; PCR, polymerase chain reaction; pEGFR, phosphorylated epidermal growth factor receptor; RIPA, radioimmunoprecipitation assay; SCI, spinal cord injury; TNFα, tumor necrosis factor alpha.

## Competing interests

The authors declare they have no competing interests.

## Authors’ contributions

WSQ, DST, WW and JGL participated in the design of the experiments. WSQ, DST, ZBG and JF carried out the experiments, acquired and interpreted the data. WSQ, MJX and WW were involved in drafting the manuscript. QZ, ZYY and HQZ provided technical support during the experiments. All authors read and approved the final manuscript.

## Supplementary Material

Additional file 1 **Table S1.** Detailed information of reagents used in the present study. **Table 2:** Detailed PCR procedure used in the present study. **Figure S1:** Statistic comparison of CD11b and pEGFR expression in BV2 cells (corresponding to figure 1D). Control was taken as 100%. OD of tested proteins was normalized to OD of β-actin and its corresponding control. n = 5. ^#^, *P* < 0.01, versus control; *, *P* < 0.01, versus LPS-treated group. It demonstrates that LPS-induced elevation of CD11b **(A)**, pEGFR **(B)**, EGFR **(C)** and pEGFR/EGFR ratio **(D)**, all of which were downregulated by C225 or AG1478 equivalently. **Figure S2:** Statistic comparison of mRNA expression (corresponding to figure 2A). Control was taken as 100%. Target gene expression was normalized versus GAPDH and its corresponding control. n = 5. *, *P* < 0.05, versus LPS-treated group. It demonstrates that LPS induced a rapid but persistent elevation of IL-1β **(A)** and TNFα **(B)** mRNA expression in primary microglias, which can be reduced by pretreatment of C225 or AG1478. **Figure 3:** Statistic comparison of mRNA expression (corresponding to figure 3C/D). Control was taken as 100%. Target gene expression was normalized versus GAPDH and its corresponding control. n = 5. #, *P* < 0.05, versus control. *, *P* < 0.05, versus LPS-treated group. Primary microglias were treated by selective inhibitors of the MAPK pathway (SB203580 for p38, U0126 for Erk1/2 and SP600125 for JNK) 30 min before LPS treatment separately, each was found to reduce the LPS-induced mRNA expression of IL-1β and TNFα at 3 h after LPS stimulation, to different degrees. **Figure 4:** Semi-quantitative comparison of protein expression after SCI (corresponding to figure 4A). Sham was taken as 100%. OD of tested proteins was normalized to OD of β-actin and its corresponding control. n = 5. *, *P* < 0.05, versus sham. Time-dependent analysis demonstrates that expression of pEGFR is upregulated during 0.25 d and 14 d after SCI, with peak at 1 d after SCI; expression of EGFR is reduced at 1 d after SCI, however, upregulated at 3 d and 7 d; and, the pEGFR/EGFR ratio is elevated during 0.25 d and 7 d after SCI, with peak at 1 d after SCI. **Figure 5:** Semi-quantitative comparison of protein expression after treatment to SCI rats (corresponding to figure 5). Sham was taken as 100%. OD of tested proteins was normalized to OD of β-actin and its corresponding control. n = 5. #, *P* < 0.05, versus sham; *, *P* < 0.05, versus SCI. It demonstrates that SCI induces overexpression of pEGFR/p-Erk/p-JNK/p-p38 (1 d after SCI, **A-D**) and IL-1β/ TNFα (3 d after SCI, **E/F**); except JNK, all others can be partly attenuated by either C225 or AG1478. **Figure 6:** Semi-quantitative comparison of protein expression (corresponding to figure 6C). Fluorescent staining was performed on SCI tissues, followed by IOD analysis with Image J, which **(A and B)** demonstrates that SCI induced fierce elevation of CD11b and GFAP expression, the markers for microglia and astrocyte respectively, at day 7 after SCI; all of which are reduced by 7 d treatment with either C225 or AG1478. Those findings have been supported by western blot analysis, provided in C1 and C2. n = 5. #, *P* < 0.05, versus sham; *, *P* < 0.05, versus SCI. Click here for file
