# Supplementary material for: Inhibition of EGFR/MAPK signaling reduces microglial inflammatory response and the associated secondary damage in rats after spinal cord injury
Source: J Neuroinflammation. 2012 Jul 23;9:178. doi: 10.1186/1742-2094-9-178 (PMC3418570; doi:10.1186/1742-2094-9-178)
Supplement: Additional file 1 — Table S1. Detailed information of reagents used in the present study. Table 2: Detailed PCR procedure used in the present study. Figure S1: Statistic comparison of CD11b and pEGFR expression in BV2 cells (corresponding to figure 1D). Control was taken as 100%. OD of tested proteins was normalized to OD of β-actin and its corresponding control. n = 5. #, P < 0.01, versus control; *, P < 0.01, versus LPS-treated group. It demonstrates that LPS-induced elevation of CD11b (A), pEGFR (B), EGFR (C) and pEGFR/EGFR ratio (D), all of which were downregulated by C225 or AG1478 equivalently. Figure S2: Statistic comparison of mRNA expression (corresponding to figure 2A). Control was taken as 100%. Target gene expression was normalized versus GAPDH and its corresponding control. n = 5. *, P < 0.05, versus LPS-treated group. It demonstrates that LPS induced a rapid but persistent elevation of IL-1β (A) and TNFα (B) mRNA expression in primary microglias, which can be reduced by pretreatment of C225 or AG1478. Figure 3: Statistic comparison of mRNA expression (corresponding to figure 3C/D). Control was taken as 100%. Target gene expression was normalized versus GAPDH and its corresponding control. n = 5. #, P < 0.05, versus control. *, P < 0.05, versus LPS-treated group. Primary microglias were treated by selective inhibitors of the MAPK pathway (SB203580 for p38, U0126 for Erk1/2 and SP600125 for JNK) 30 min before LPS treatment separately, each was found to reduce the LPS-induced mRNA expression of IL-1β and TNFα at 3 h after LPS stimulation, to different degrees. Figure 4: Semi-quantitative comparison of protein expression after SCI (corresponding to figure 4A). Sham was taken as 100%. OD of tested proteins was normalized to OD of β-actin and its corresponding control. n = 5. *, P < 0.05, versus sham. Time-dependent analysis demonstrates that expression of pEGFR is upregulated during 0.25 d and 14 d after SCI, with peak at 1 d after SCI; expression of EGFR is reduced at 1 d aft [file 1742-2094-9-178-S1.doc]

**Supplementary materials：**

Supplementary table 1: Detailed information of reagents used in the present study

| Method | Regent | Usage and Source |
| --- | --- | --- |
| Fluorescent staining | CD11b mouse mAb | 1:50, BD Biosciences, San Jose, CA, USA |
|  | GFAP rabbit mAb | Chemicon, Temecula, CA, USA |
|  | pEGFR rabbit mAb | 1:100, Cell Signaling Technology, Beverly, MA, USA |
|  | Avidin-FITC | 1:67, Boster, Hubei, China |
|  | FITC conjugated anti-mouse IgG | 1:200, Jackson Immuno-Research, West Grove, PA, USA |
|  | Cy3 conjugated anti-rabbit IgG | 1:300, Jackson Immuno-Research, |
|  | DAPI | 5μg/ml, Sigma-Aldrich |
| Western blot | pEGFR rabbit mAb | 1:1000, Cell Signaling Technology |
|  | EGFR rabbit pAb | 1:200, Santa-Cruz Biotechnology, Santa Cruz, CA, USA |
|  | Phospho-Erk1/2 rabbit mAb | 1:1000, Cell Signaling Technology, Beverly, MA, USA |
|  | Phospho-JNK mouse mAb | 1:800, Cell Signaling Technology |
|  | Phosph-p38 mouse mAb | 1:800, Cell Signaling Technology |
|  | IL-1β and TNFα mouse pAb | 1:400, Boster, Hubei, China |
|  | β-actin mouse mAb | 1:2000, Sigma, St Louis, MO, USA |
|  | HRP-conjugated IgG | 1:3000, Pierce, Rockford, IL, USA |
|  | RIPA lysis buffer | Pierce |
|  | BCA kit | Pierce |
|  | ECL kit | Pierce |
| Cell treatment | C225 | 20nM, Merck KGaA, Darmstadt, Germany |
|  | LPS | 1µg/ml, Sigma-Aldrich |
|  | AG1478 | 10μM, Sigma-Aldrich |
|  | SB203580 | 10μM, Sigma-Aldrich |
|  | U0126 | 10μM, Sigma-Aldrich |
|  | SP600125 | 10μM, Sigma-Aldrich |
|  | high glucose DMEM containing | HyClone, Logan, UT, USA |
|  | fetal bovine serum | HyClone |
| others | MagExtractor | Toyobo Co. Ltd, Japan |
|  | ReverTra Ace | Toyobo |
|  | hot-start PCR mix | Tiangen biotech, Beijing, China |
|  | ELISA kit for rat IL-1β and TNFα | R&D Systems, Minneapolis, MN, USA |
|  | BDA | Neurotrace®, Molecular probes, Oregon, USA |

Supplementary table 2: Detailed PCR procedure used in the present study

| Target | Primers and reaction | Length of product |
| --- | --- | --- |
| TNFα | Sense: 5’-ACCGTCAGCCGATTTGCC  Antisense: 5’-ACACGCCAGTCGCTTCAC  Reaction: 94 °C 10 min, (degeneration 45 s at 94 °C, annealing 30 s at 55 °C, extension 30 s at 72 °C)×35 cycles, 72 °C 10 min. | 295bp |
| IL-1β | Sense: 5’-TGTGATGTTCCCATTAGAC  Antisense: 5’-AATACCACTTGTTGGCTTA  Reaction: 94 °C 10 min, (degeneration 45 s at 94 °C, annealing 30 s at 58 °C, extension 30 s at 72 °C)×35 cycles, 72 °C 10 min. | 131bp |
| GAPDH | Sense: 5’-TGCCCACCAGAACATCAT  Antisense: 5’-TAGCCATATTCGTTGTCGTA  Reaction: 94 °C 10 min, (degeneration 45 s at 94 °C, annealing 30 s at 52 °C, extension 30 s at 72 °C)×35 cycles, 72 °C 10 min. | 357bp |

Supplementary figure 1:


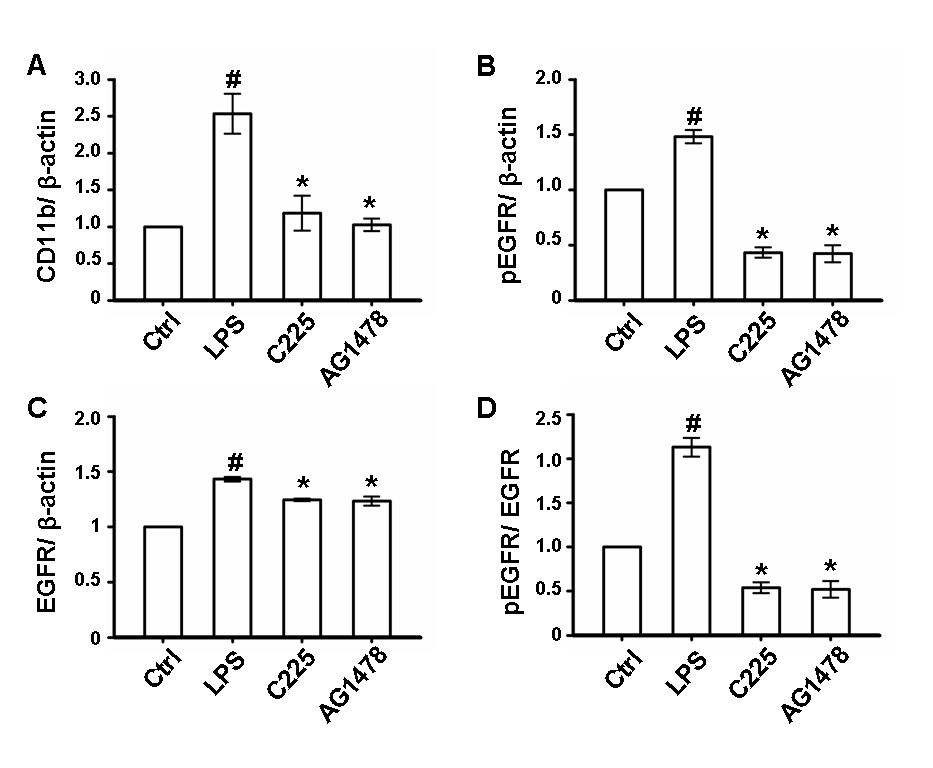


Fig 1: Statistic comparison of CD11b and pEGFR expression in BV2 cells (corresponding to figure 1D). Control was taken as 100%. OD of tested proteins was normalized to OD of β-actin and its corresponding control. *n*=5. ^#^, *P*＜0.01, vs. control; *, *P*＜0.01, vs. LPS-treated group. It demonstrates that LPS induced elevation of CD11b (A), pEGFR (B), EGFR (C) and pEGFR/EGFR ratio (D), all of which were downregulated by C225 or AG1478 equivalently.

Supplementary figure 2:


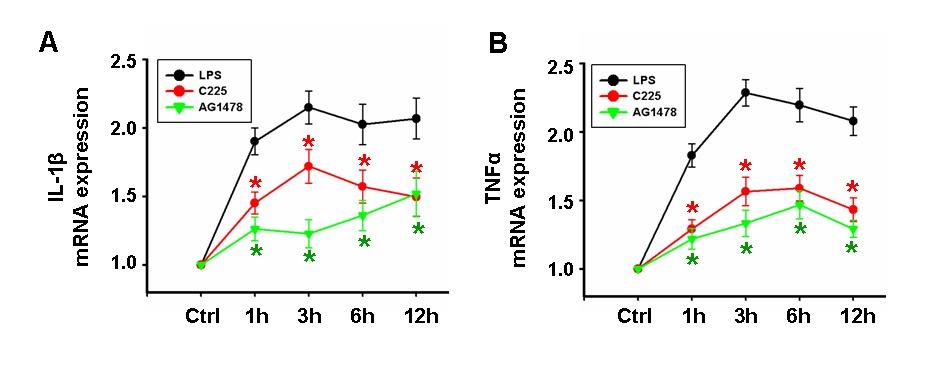


Fig 2: Statistic comparison of mRNA expression (corresponding to figure 2A). Control was taken as 100%. Target gene expression was normalized versus GAPDH and its corresponding control. *n*=5. *, *P*＜0.05, vs. LPS-treated group. It demonstrates that LPS induced a rapid but persistent elevation of IL-1β (A) and TNFα (B) mRNA expression in primary microglias, which can be reduced by pre-treatment of C225 or AG1478.

Supplementary figure 3


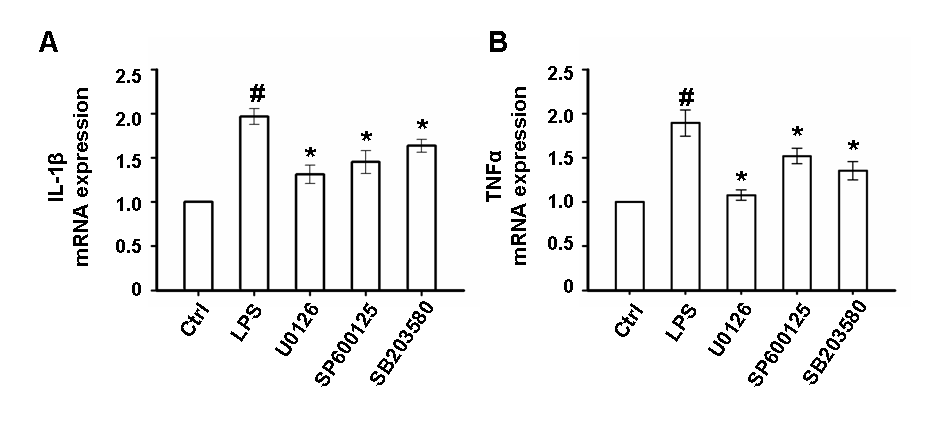


Fig 3: Statistic comparison of mRNA expression (corresponding to figure 3C/D). Control was taken as 100%. Target gene expression was normalized versus GAPDH and its corresponding control. n=5. #, *P*＜0.05, vs. control. *, *P*＜0.05, vs. LPS-treated group. Primary microglias were treated by selective inhibitors of the MAPK pathway (SB203580 for p38, U0126 for Erk1/2 and SP600125 for JNK) 30 min before LPS treatment separately, each was found to reduce the LPS induced mRNA expression of IL-1β and TNFα at 3 h after LPS stimulation, to different degrees.

Supplementary figure 4


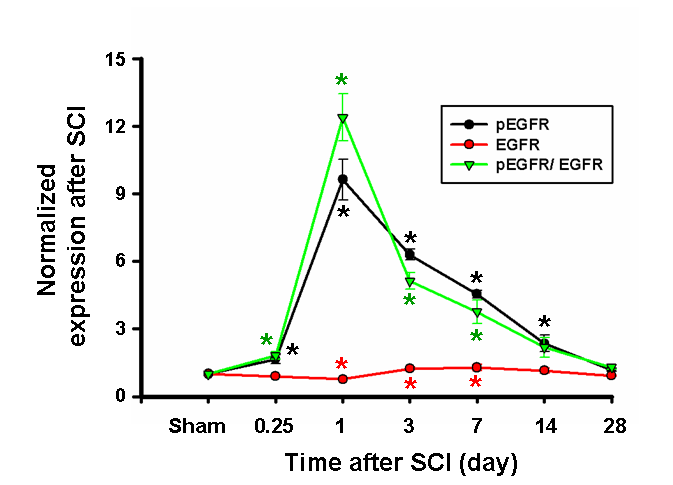


Fig 4: Semi-quantitative comparison of protein expression after SCI (corresponding to figure 4A). Sham was taken as 100%. OD of tested proteins was normalized to OD of β-actin and its corresponding control. *n*=5. *, *P*＜0.05, vs. sham. Time-dependent analysis demonstrates that expression of pEGFR is upregulated during 0.25 d and 14 d after SCI, with peak at 1 d after SCI; expression of EGFR is reduced at 1 d after SCI, however, upregulated at 3 d and 7 d; and, the pEGFR/EGFR ratio is elevated during 0.25 d and 7 d after SCI, with peak at 1 d after SCI.

Supplementary figure 5:


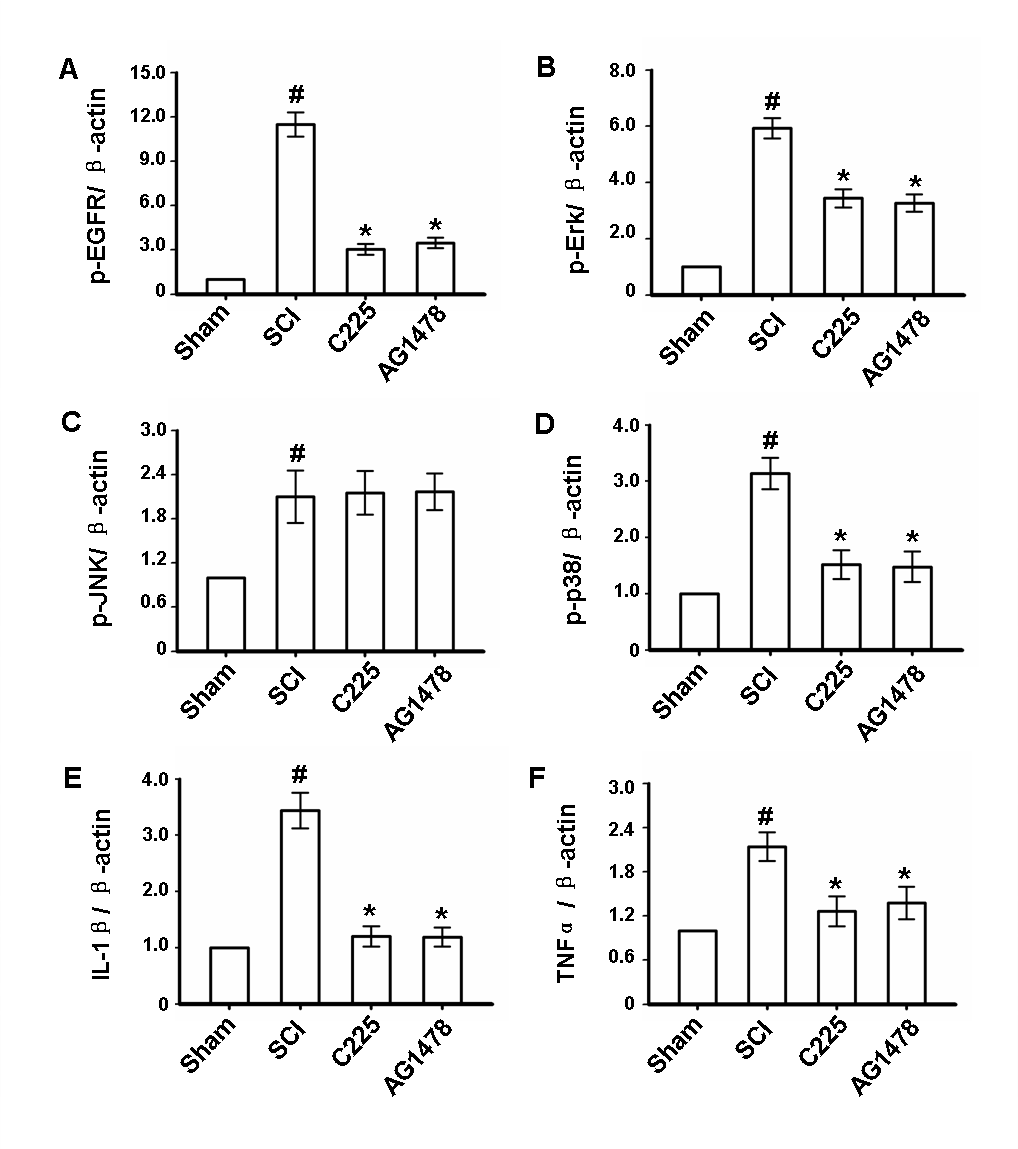


Fig 5: Semi-quantitative comparison of protein expression after treatment to SCI rats (corresponding to figure 5). Sham was taken as 100%. OD of tested proteins was normalized to OD of β-actin and its corresponding control. *n*=5. #, *P*＜0.05, vs. sham; *, *P*＜0.05, vs. SCI. It demonstrates that SCI induces over-expression of pEGFR/ p-Erk/ p-JNK/ p-p38 (1 d after SCI, A-D) and IL-1β/ TNFα (3 d after SCI, E/F); except JNK, all others can be partly attenuated by either C225 or AG1478.

Supplementary figure 6:


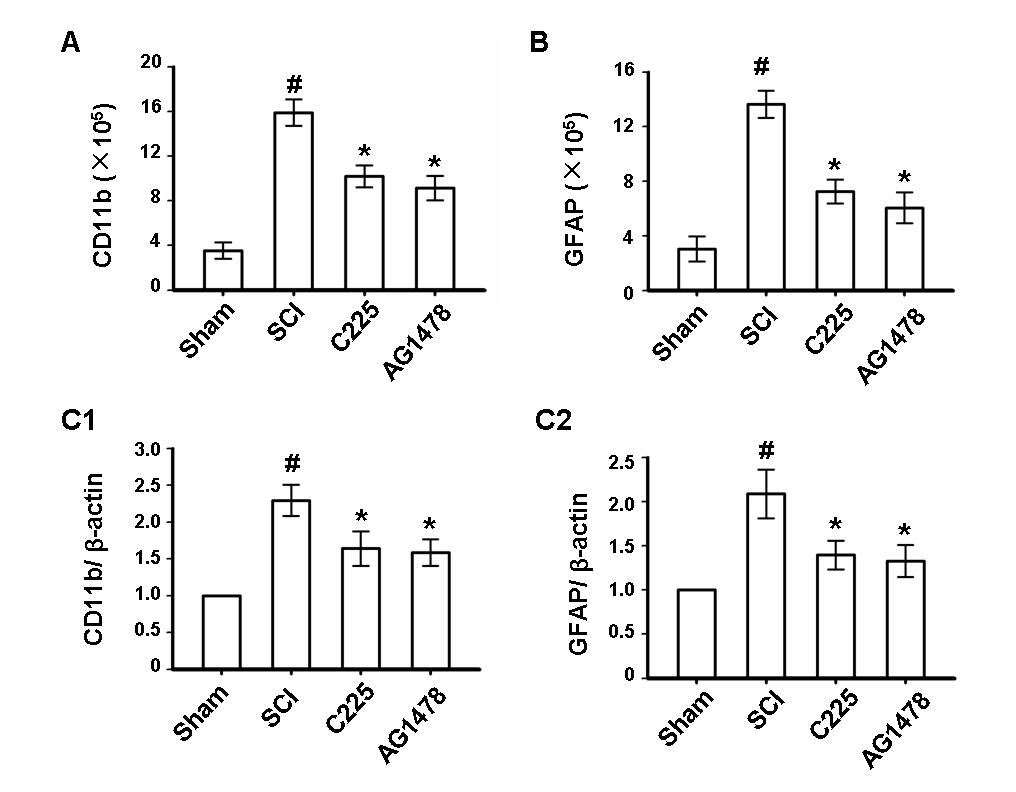


Fig 6: Semi-quantitative comparison of protein expression (corresponding to figure 6C). Fluorescent staining was performed on SCI tissues, followed by IOD analysis with Image J, which (A and B) demonstrates that SCI induced fierce elevation of CD11b and GFAP expression, the markers for microglia and astrocyte respectively, at day 7 after SCI; all of which are reduced by 7 d treatment with either C225 or AG1478. Those findings have been supported by western blot analysis, provided in C1 and C2. *n*=5. #, *P*＜0.05, vs. sham; *, *P*＜0.05, vs. SCI.
